# Comparing Antepartum and Postpartum Opioid-Related Maternal Deaths in the State of Michigan From 2007 to 2015

**DOI:** 10.7759/cureus.48690

**Published:** 2023-11-12

**Authors:** Manesha Putra, Micaela Roy, Vanessa Nienhouse, Kara Patek, Robert Sokol

**Affiliations:** 1 Obstetrics and Gynecology, University of Colorado School of Medicine, Denver, USA; 2 Obstetrics and Gynecology, Wayne State University School of Medicine, Detroit, USA

**Keywords:** pregnancy-associated not related, postpartum, antepartum, maternal mortality, pregnancy, opioid use disorder

## Abstract

Objective

Opioid use disorder (OUD) continues to be a leading cause of maternal death in the United States. The impact of OUD on pregnancy has dramatically grown in recent years, with OUD-related maternal deaths between 2007 and 2016 nearly doubling. However, the characteristics of pregnancy-associated-not-related (PANR) deaths from opioid overdose are not well understood. Specifically, the timing of OUD-related maternal deaths relative to the partum periods has not been fully described. In this study, we aimed to better characterize high-risk time periods for people with OUD, with the goal of elucidating factors that may contribute to opioid-related PANR deaths.

Methods

In this retrospective cohort study, we analyzed the Michigan Department of Health and Human Services Maternal Mortality Surveillance Program database from 2007 to 2015 to investigate the temporal trends in opioid-related PANR deaths.

Results

There was an over fourfold increase in opioid-related PANR from 2007 to 2015 and a maternal mortality ratio of 23.0 per 100,000 births attributable to opioid-related PANR deaths. Ante- and postpartum opioid-related PANR deaths shared similar demographic distribution, were associated with polysubstance use, and had low rates of medication-assisted treatment (MAT). Most opioid-related PANR deaths occurred at a steady rate during the postpartum period. Only 3.6% of people who died in the postpartum period were uninsured, compared to 42.1% of people who died in the antepartum period.

Conclusion

Though ante and postpartum deaths share many characteristics, our study revealed key distinctions that can help better inform the care of pregnant patients with OUD.

## Introduction

Opioid use disorder (OUD) continues to be a major concern in pregnancy and is increasingly seen during delivery hospitalization [1]. People with OUD during pregnancy are at higher risk of preterm delivery, fetal growth restriction, and stillbirth and are four times more likely to die during their hospital stay [2,3]. In recent years, the impact of OUD on maternal health has grown. From 2005 to 2014, there was a nearly three-fold increase in pregnancy-associated mortality ratio related to opioid use, and the number of OUD-related maternal deaths between 2007 and 2016 more than doubled [4,5]. Furthermore, overdose has been cited as a leading cause of maternal mortality in several states, including Colorado, California, Virginia, Massachusetts, and Illinois [6-10].

Understanding the characteristics and timing (i.e., antepartum vs. intrapartum vs. postpartum) of maternal death in cases of OUD may help inform health interventions and resource allocation to reduce maternal mortality [11]. Previous studies have begun to investigate the trends in maternal mortality during and after pregnancy. Overwhelmingly, studies have shown that most OUD-related maternal deaths happen postpartum, though significant mortality does occur in the antepartum and intrapartum periods. A study from 2013 demonstrated that 42% of pregnancy deaths due to OUD happened during the immediate postpartum period (one to 42 days after delivery), followed by intrapartum at 22%, antepartum at 21% and late postpartum period at 15% (42 days to one year after delivery) [12]. A Massachusetts study of both fatal and non-fatal overdose showed that overdose events were least prevalent in the third trimester, and most overdoses occurred 7-12 months postpartum [6]. Despite growing interest in opioid-related deaths in this patient population, more research is needed to fully understand opioid-related pregnancy-associated-not-related (PANR) deaths in the antepartum and postpartum periods. Better identifying and characterizing high-risk time periods for opioid-related PANR is essential in combating maternal mortality in the U.S.

The objectives of our study were to characterize high-risk time periods of opioid-related PANR deaths. Using the Michigan Department of Health and Human Services Maternal Mortality Surveillance Program database from 2007 to 2015, we compared the characteristics of opioid-related PANR deaths in antepartum and postpartum periods. By investigating the temporal trends of opioid-related PANR, we aim to further inform programs for the treatment and prevention of maternal mortality due to OUD.

## Materials and methods

We conducted a retrospective cohort study of all PANR deaths using the Michigan Department of Health and Human Services Maternal Mortality Surveillance Program database from 2007-2015. This program was established to improve the quality of maternal death data in Michigan and to bring awareness to the importance of preventing death among women during or within one year of pregnancy. The state mandates reporting of all records associated with maternal death, and subsequently, a multidisciplinary team reviewed the process to determine causes of death and to make policy recommendations to prevent future deaths. This study was considered exempt from full review by the Institutional Review Board (Michigan Department of Health and Human Services Institutional Review Board for the Protection of Human Research Subjects, Lansing, Michigan).

We identified all PANR deaths within the database, which were defined as death occurring during pregnancy or within one year of the end of pregnancy, but not related to pregnancy. Inclusion criteria for the study are all PANR deaths in the state of Michigan from 2007 to 2015, deemed by the surveillance program to be related to OUD. The available records of each case of death, including prenatal and delivery records, birth certificates, death certificates, and police reports, were systematically reviewed. We collected information on the year of death, region of death, manner of death, patient demographics, delivery outcomes, comorbidities, and urine and post-mortem toxicology studies. Descriptive statistics were used to characterize baseline demographic data and temporal trends for opioid-related PANR deaths. Univariate analyses were performed to compare each variable characteristic of antepartum vs. postpartum opioid-related PANR deaths, with the Mann-Whitney U test for continuous variables and the Chi-squared test for categorical variables. A multivariable logistic regression model was built using maternal age, race/ethnicity, insurance status, and exposure to medication-assisted treatment (MAT) to determine which baseline characteristics were most correlated to opioid-related PANR deaths in the antepartum vs. postpartum period (postpartum period was used as the reference group). Differences in the timing of death between individuals with and without MAT was assessed by Kaplan-Meir curves and the log-rank test.

## Results

**Figure 1:**
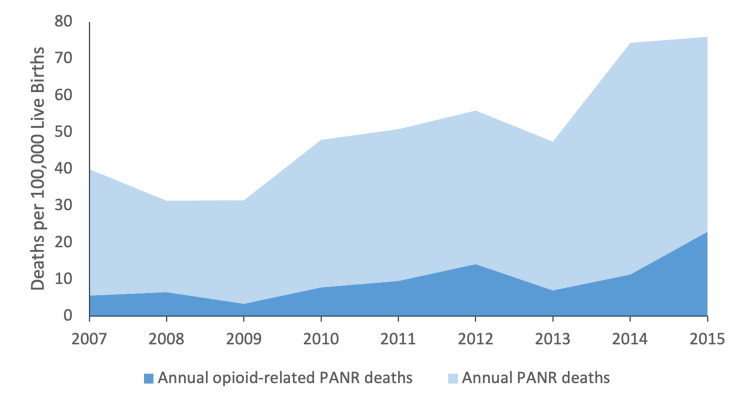
The trend in annual opioid-related pregnancy-associated-not-related deaths vs. pregnancy-associated-not-related deaths PANR = pregnancy-associated-not-related

**Figure 2:**
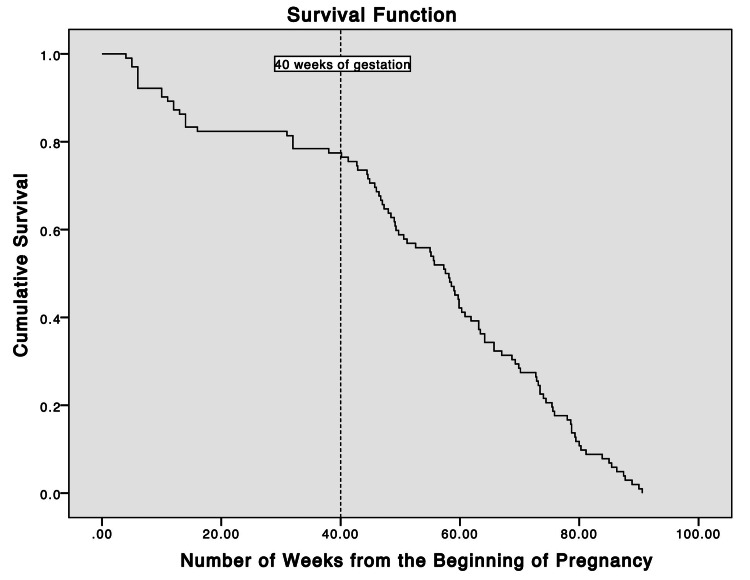
Survival plot for opioid-related pregnancy-associated-not-related deaths

**Figure 3:**
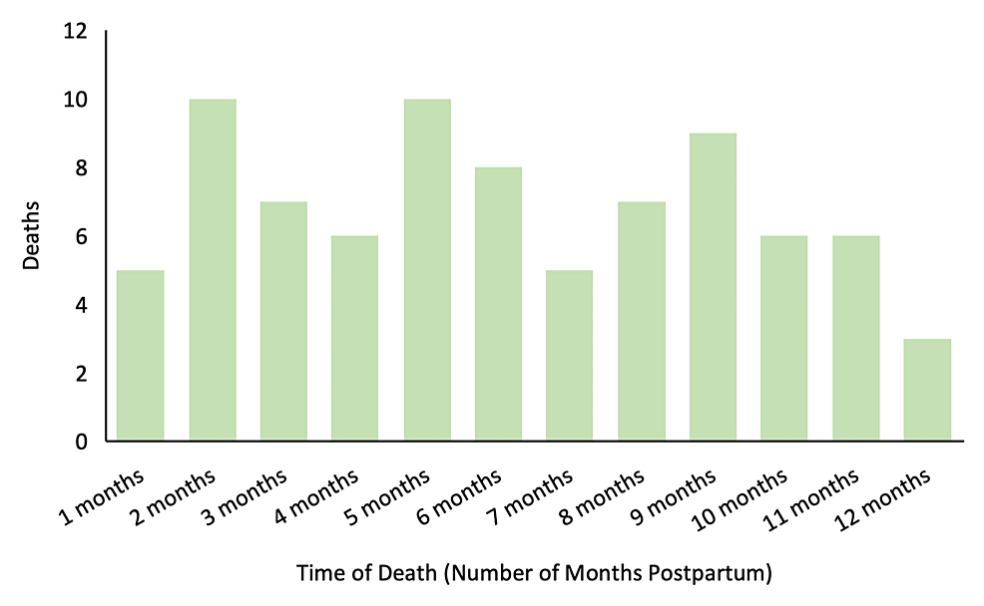
Distribution of opioid-related pregnancy-associated-not-related deaths

From January 2007 to December 2015, 424 PANR deaths were identified in the state of Michigan. A total of 102/424 (24.1%) PANR deaths were related to OUD, of which 19/102 (19.6%) occurred during the antepartum period, and 83/102 (81.4%) occurred during the postpartum period. During the study period, the overall opioid-related PANR and non-opioid-related PANR mortality ratios were 9.7 and 30.8 deaths per 100,000 live births, respectively. There was a steady increase in opioid-related PANR deaths throughout the study years, with the maternal mortality ratio peaking at 23.0 per 100,000 live births in 2015 (Figure 1). There was a higher rate of death during the postpartum period relative to the antepartum period, as shown in Figure 2. The rate of death throughout the postpartum period was largely constant (Figure 3).

**Figure 4:**
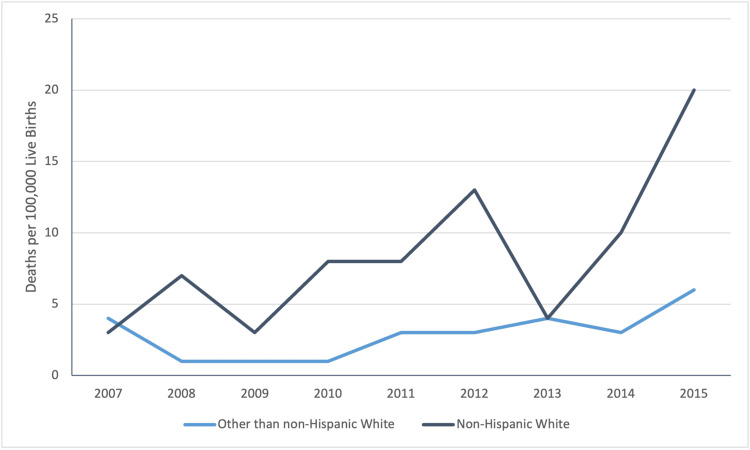
The trend in annual opioid-related pregnancy-associated-not-related deaths in non-Hispanic White vs. other

**Figure 5:**
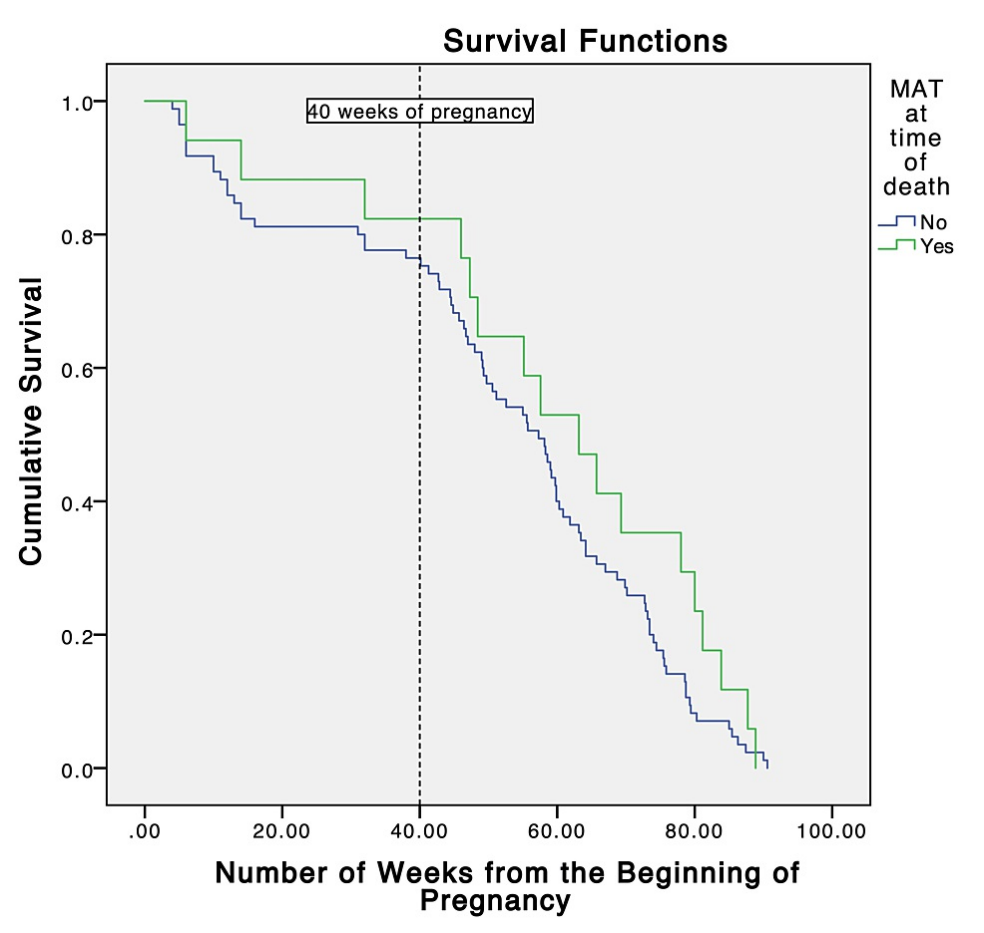
Survival plot of patients treated with and without medication-assisted treatment at the time of death The logrank test was performed to determine statistical significance (p=0.203) MAT = medication-assisted treatment

The characteristics of total, antepartum, and postpartum opioid-related PANR deaths are listed in Table 1. There were no significant differences in age, gravidity, parity, racial distributions, or pre-pregnancy comorbidities between postpartum and antepartum PANR deaths. People with antepartum opioid-related PANR deaths had significantly fewer antenatal visits than those with postpartum opioid-related PANR deaths, with only 2/19 (0.1%) having any antenatal visits. A multivariable logistic regression analysis revealed that uninsured status was significantly associated with antepartum opioid-related PANR death (Table 2). However, maternal age, race/ethnicity, and exposure to MAT were not significantly linked to opioid-related PANR mortality. Temporal trends of opioid-related PANR deaths in racial minorities relative to non-Hispanic whites varied, as shown in Figure 4. Although MAT was not significantly linked to opioid-related PANR mortality, there were differences in the survival trends between patients with and without MAT at the time of death (Figure 5).

**Table 1:** Characteristics of total, antepartum, and postpartum opioid-related pregnancy-associated-not-related deaths Numbers are displayed in either median (inter-quartile range) or n (%) format. Statistical significance is denoted by *. Pre-pregnancy comorbidities include obesity, chronic hypertension, pre-gestational diabetes, human immunodeficiency virus (HIV), and hepatitis C. GA = gestational age; N/A = not applicable; MAT = medication assisted treatment

Characteristics	Period of death			p-value
	Total (n=102)	Antepartum (n=19)	Postpartum (n=83)	
Maternal age (years)	28 (24 - 33)	26 (23 - 33)	28 (24 - 33)	0.781
Gravidity	3 (2 - 5)	3 (2 - 4.25)	3 (2 - 5)	0.862
Parity	2 (1 - 3)	2 (1.5 - 3.25)	2 (1 - 3)	0.014*
GA at death (weeks)	14.0 (6.0 - 31.0)	14.0 (6.0 - 31.0)	N/A	N/A
Week postpartum	23.4 (11.1 - 38.0)	N/A	23.4 (11.1 - 38.0)	N/A
Race				0.538
Non-Hispanic White	76 (74.5)	16 (84.2)	60 (72.3)	
Non-Hispanic Black	18 (17.6)	3 (15.8)	15 (18.1)	
Other	8 (7.8)	0 (0)	8 (9.6)	
Insurance status				<0.001*
Public insurance	78 (76.5)	10 (52.6)	68 (81.9)	
Private insurance	13 (12.7)	1 (5.3)	12 (14.5)	
Uninsured	11 (10.8)	8 (42.1)	3 (3.6)	
Employment status				0.516
Employed	60 (58.8)	13 (68.4)	47 (56.6)	
Unemployed	39 (38.2)	6 (31.6)	33 (39.8)	
Student	3 (2.9)	0 (0)	3 (3.6)	
Education status				0.288
High school/less	76 (74.5)	10 (52.6)	66 (79.5)	
Some college/more	26 (25.5)	9 (47.4)	17 (20.5)	
Number of prenatal visits	7 (0 - 10)	0 (0 - 0)	8 (4 - 8)	0.001*
Pre-pregnancy comorbidities				0.118
Yes	39 (38.2)	4 (21.1)	48 (57.8)	
No	63 (61.8)	15 (78.9)	35 (42.2)	
Pregnancy MAT				0.514
Yes	18 (17.6)	2 (10.5)	16 (19.3)	
No	84 (82.4)	17 (89.5)	67 (80.7)	
MAT at death				0.733
Yes	17 (16.7)	2 (10.5)	15 (18.1)	
No	85 (83.3)	17 (89.5)	68 (81.9)	

**Table 2:** Multivariable logistic regression comparison of opioid-related pregnancy-associated-not-related deaths in the antepartum vs. postpartum periods The postpartum period was used as the reference group. Statistical significance is denoted by *. MAT = medication-assisted treatment; CI = confidence interval; OR = odds ratio.

Predictors	OR	95% CI	p-value
Maternal age	1.09	0.84 - 6.03	0.144
Race/ethnicity	2.25	0.84 - 6.03	0.10
Uninsured status	0.20	0.09 - 0.46	<0.001*
Exposure to MAT	0.06	0.92 - 45.56	0.061

**Figure 6:**
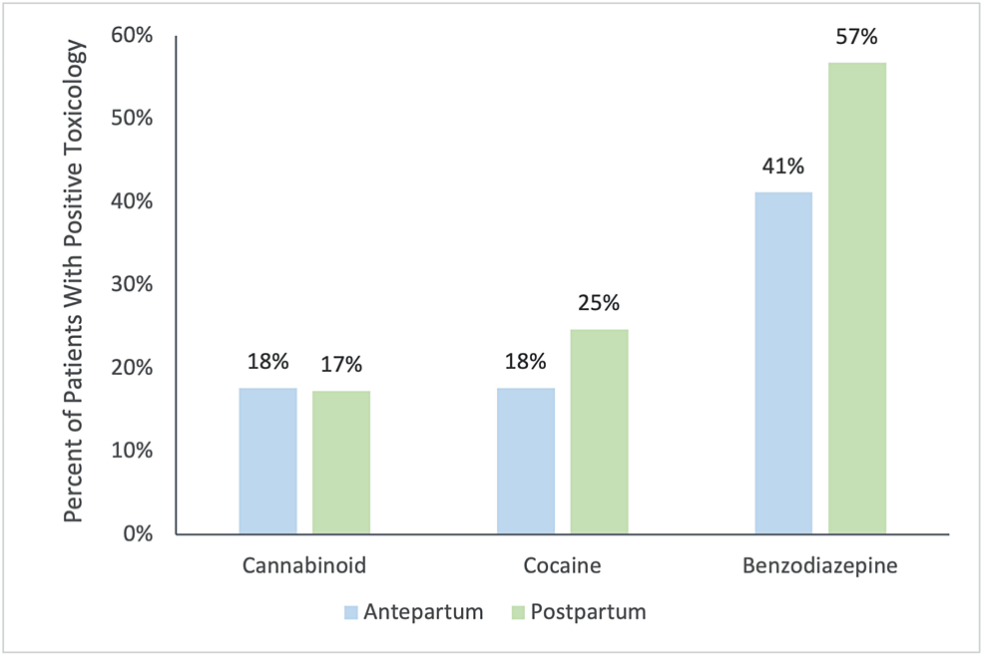
Non-opiate drugs detected at autopsy

Of the 102 people with opioid-related PANR deaths, 77/102 (75.5%) deaths were determined to be accidental deaths, 2/102 (2.0%) were suicides, and 23/102 (22.5%) were indeterminate. There was no difference in the distribution of manner of death between antepartum and postpartum deaths (p=0.514). An autopsy was performed in 100/102 (98.0%) of deaths, and toxicology was performed in 98/102 (96.0%) of the cases. Benzodiazepine was found in 53/98 (54.0%) of autopsy toxicology studies, followed by cocaine in 23/98 (23.5%) studies. A comparison of non-opioid drugs detected from autopsy toxicology in antepartum opioid-related PANR deaths relative to postpartum opioid-related PANR deaths is shown in Figure 6. 

In terms of geographic area, most opioid-related PANR deaths occurred in region 10, which includes the three most populous counties in Michigan. The proportion of opioid-related PANR deaths in region 10 was higher than the proportion of live births in this region (52/102, 51.0% vs. 423,832/1,046,699, 40.5%, p=0.03). 

## Discussion

Our study showed a steady increase in opioid-related PANR deaths in the state of Michigan from 2007-2015, with an over four-fold increase over the study period. At the end of the study period, there was a maternal mortality ratio of 23.0 per 100,000 births attributable to opioid-related PANR deaths. This finding echoes previous studies that demonstrate increasing opioid-related PANR deaths in the United States over this time period [3-5]. However, our maternal mortality ratio for the state of Michigan was over twice as high as a previous statewide report within a similar time period (23.0 vs. 11.7) [4]. 

Ante- and postpartum opioid-related PANR deaths shared largely similar demographic distribution. We also see a low rate of MAT in both time frame of deaths, similar to previous reports [4,6]. This rate reflects the current low rate of MAT in patients with OUD, which is estimated to be slightly under 20% [13]. Although we did not see significant differences between deaths in patients with or without MAT, there were interesting differences in the survival plots of these two groups at the time of death, with higher cumulative survival in MAT patients (Figure 5). This supports previous studies that suggest that MAT significantly reduces overdose rates in the early postpartum period, highlighting the potential role of MAT in preventing opioid-related PANR deaths [6].

Most antepartum and postpartum opioid-related PANR deaths were associated with polysubstance use. These results reflect previous research, which suggests that most people with OUD use one or more additional substances. The most commonly found substance on toxicology was benzodiazepine, which dramatically increases overdose risk when combined with opiates (Figure 6). Thus, these findings reiterate the need to reduce co-prescription of these medications and increase awareness of their dangerous effects. 

The majority of opioid-related PANR deaths in this study occurred during the postpartum period, adding to an extensive body of literature that suggests that increased intervention for OUD is needed during this time period [4,6,8]. While previous reports have shown that most opioid-related PANR deaths occur after six weeks postpartum [4], our data demonstrated a steady rate of deaths across the entire postpartum period, similar to Metz et al. [8] (Figure 3). This indicates the need to implement consistent interventions throughout the first year postpartum. Future studies that investigate opioid-related PANR deaths over a port-partum period longer than one year could provide additional insight into maternal risks over time. This, along with the fact that uninsured people accounted only for 3.6% of postpartum opioid-related PANR deaths, strongly suggests that expanding access to insurance during the postpartum period alone is unlikely to be sufficient to effectively reduce opioid-related PANR deaths.

As opposed to in the postpartum period, 42.1% of antepartum opioid-related PANR deaths occurred in uninsured patients. The association between insurance status and antepartum death remained even after controlling for race/ethnicity, maternal age, and exposure to MAT. Furthermore, all the antepartum deaths occurred among people who did not have prenatal care. Although causation cannot be assumed, this relationship may indicate that obstetric-specific care could reduce opioid-related PANR deaths during ante- and postpartum periods. Unfortunately, previous studies have shown a low rate of postpartum care, even among people with pregnancy complications [14-16]. While the American College of Obstetricians and Gynecologists (ACOG) has reinforced the importance of optimization of postpartum care, the impact of this statement remains to be seen. Finding ways to increase postpartum and antepartum care in people with OUD could represent a critical first step in reducing opioid-related PANR deaths. 

Classically, the opioid epidemic has mostly been attributed to White, middle-class, suburban, and rural communities [17,18]. While our data reflects the largely white demographics of Michigan, Black and minority Americans are slightly overrepresented in our study. 17.6% of opioid-related PANR deaths occurred in Black Americans, though only 14.2% of the population in 2010 identified as Black [19]. People who identified as American Indian or Alaska Native were overrepresented nearly fivefold, accounting for 2.9% of opioid-related PANR deaths, though only 0.6% of the population identified as American Indian or Alaska Native in 2010 [19]. With these findings in mind, we hope to call for an equitable approach to solutions to reduce opioid-related PANR deaths that do not solely focus on white communities. We fear that if the efforts to combat the opioid epidemic are not distributed to all pregnant people equitably, we will merely shift the death to marginalized communities.

Our study is subject to several limitations. Because our data is limited to mortality data, we are unable to measure the impact of MAT in reducing opioid-associated PANR deaths. Additionally, despite the Michigan mandate to report OUD-related PANR deaths, it is possible that some healthcare encounters were not reported. Finally, repeating this study with a multi-state data set could illuminate additional trends by increasing sample size, especially in the antepartum period. 

## Conclusions

In conclusion, ante- and postpartum opioid-related PANR deaths share demographic similarities, as well as associations with polysubstance use and low rates of MAT. However, key differences remain. Notably, a large proportion of antepartum opioid-related PANR deaths occur in uninsured patients. Additionally, the majority of opioid-related PANR happen at a steady rate during the postpartum period. These trends may help to tailor comprehensive programs for people with OUD during the pregnancy and postpartum periods globally.

Our study supports the following: 1) access to initiate prenatal care is essential in the antepartum period, 2) along with improvement in access to insurance during the postpartum period, efforts to increase visits and healthcare interface needs to be performed, 3) MAT needs to be made accessible throughout pregnancy periods, and 4) while traditionally seen as majority White issue, equitable programs including campaign and effort in marginalized communities is of utmost importance.
